# Glycated hemoglobin and body mass index as mediators of GLP‐1RAs and Alzheimer's disease and related dementias in patients with type 2 diabetes

**DOI:** 10.1002/alz.70161

**Published:** 2025-04-10

**Authors:** Huilin Tang, William T Donahoo, Steven T. DeKosky, Yao An Lee, Mikael Svensson, Jiang Bian, Jingchuan Guo

**Affiliations:** ^1^ Department of Pharmaceutical Outcomes and Policy University of Florida College of Pharmacy Gainesville Florida USA; ^2^ Department of Medicine University of Florida College of Medicine Gainesville Florida USA; ^3^ Department of Neurology and McKnight Brain Institute University of Florida College of Medicine Gainesville Florida USA; ^4^ Florida Alzheimer's Disease Research Center (ADRC) University of Florida Gainesville Florida USA; ^5^ Center for Drug Evaluation and Safety University of Florida Gainesville Florida USA; ^6^ Department of Health Outcomes and Biomedical Informatics College of Medicine University of Florida Gainesville Florida USA

**Keywords:** Alzheimer's disease and related dementias, body mass index, causal mediation analysis, GLP‐1RAs, glycated hemoglobin, type 2 diabetes

## Abstract

**INTRODUCTION:**

Whether reductions in glycated hemoglobin (HbA1c) levels and body mass index (BMI) mediate the association between glucagon‐like peptide‐1 receptor agonists (GLP‐1RAs) and Alzheimer's disease and related dementias (ADRD) risk is unknown.

**METHODS:**

This cohort study included 22,908 patients aged ≥ 50 years with type 2 diabetes (T2D) newly prescribed GLP‐1RA or other second‐line glucose‐lowering drugs (GLDs). Causal mediation analysis was used to estimate to what extent the effect of GLP‐1RAs on ADRD risk was attributable to lowering HbA1c or BMI.

**RESULTS:**

Compared to other GLD users, GLP‐1RA users had significant reductions in HbA1c levels by 0.16% and BMI by 0.23 kg/m^2^, along with a 26% lower ADRD risk. The direct protective effect of GLP‐1RAs on ADRD risk persisted even after accounting for HbA1c and BMI reductions, with minimal mediation effects observed through these factors.

**DISCUSSION:**

GLP‐1RAs reduce ADRD risk, largely independent of their effects on HbA1c and BMI.

**Highlights:**

- Glucagon‐like peptide‐1 receptor agonists (GLP‐1RAs) were associated with reductions in glycated hemoglobin (HbA1c) and body mass index (BMI) compared to other second‐line glucose‐lowering drugs(GLDs).
- GLP‐1RA users were associated with a 26% lower risk of Alzheimer's disease and related dementias (ADRD) than other GLD users.
- The protective effect of GLP‐1RAs against ADRD in adults with type 2 diabetes (T2D) is largely independent of their effects on HbA1c and BMI.

## 1 BACKGROUND

Alzheimer's disease and related dementias (ADRD) are progressive neurodegenerative diseases that disproportionately affect older adults, particularly those with comorbidities, such as diabetes and hypertension.^1^ These conditions pose a significant public health challenge, with approximately 6.9 million geriatric Americans living with ADRD in 2023, and this number is projected to double by 2060,^2^ underscoring the urgent need for effective prevention and treatment strategies. Glucagon‐like peptide‐1 receptor agonists (GLP‐1RAs), approved for treating type 2 diabetes (T2D), hold promise as potential strategies for preventing or treating ADRD. A systematic review and meta‐analysis of 10 observational studies involving 819,511 patients with T2D revealed that GLP‐1RAs were significantly associated with a reduced risk of all‐cause dementia.^3^

While the exact mechanisms underlying the decreased risk of ADRD associated with GLP‐1RAs remain to be fully elucidated, several hypotheses have been proposed.^4^ One potential pathway involves the ability of GLP‐1RAs to decrease glycated hemoglobin (HbA1c) and body mass index (BMI),^5^, ^6^ which are related to the risk of ADRD. Elevated glucose levels/HbA1c have emerged as risk factors for dementia, even among patients without diabetes.^7^, ^8^ Extensive studies have shown that uncontrolled blood glucose is an independent risk factor for cognitive decline, particularly in the diabetic population.^9^, ^10^ The relationship between BMI and ADRD risk is more complex and remains controversial. Generally, a higher midlife BMI was associated with an increased risk of ADRD, whereas a higher late‐life BMI was associated with a reduced risk.^11^ This paradoxical relationship underscores the need for further investigation into the role of BMI in ADRD pathogenesis. Despite cumulative evidence supporting the neuroprotective effects of GLP‐1RAs, little is known about whether HbA1c and BMI reductions mediate the association between GLP‐1RAs and ADRD risk. Causal mediation analysis is a statistical approach well‐suited to explore the possible mechanisms underlying causal treatment effects. By decomposing the total effect of an exposure on outcome into indirect (or mediated) effects through specific mediators and the remaining direct effect, it provides insight into how much of the observed treatment effect is mediated through the mediator under a counterfactual framework.^12^ Leveraging this method, we conducted a population‐based study to evaluate the mediating roles of HbA1c and BMI reductions on the association between GLP‐1RAs and ADRD. Specifically, we aimed to quantify the extent to which the decreased ADRD risk associated with GLP‐1RAs is mediated through reductions in HbA1c and BMI, thereby contributing to a deeper understanding of the pathways involved in GLP‐1RA's impact on ADRD risk in patients with T2D.

## 2 METHODS

### 2.1 Study design and data source

**FIGURE 1:**
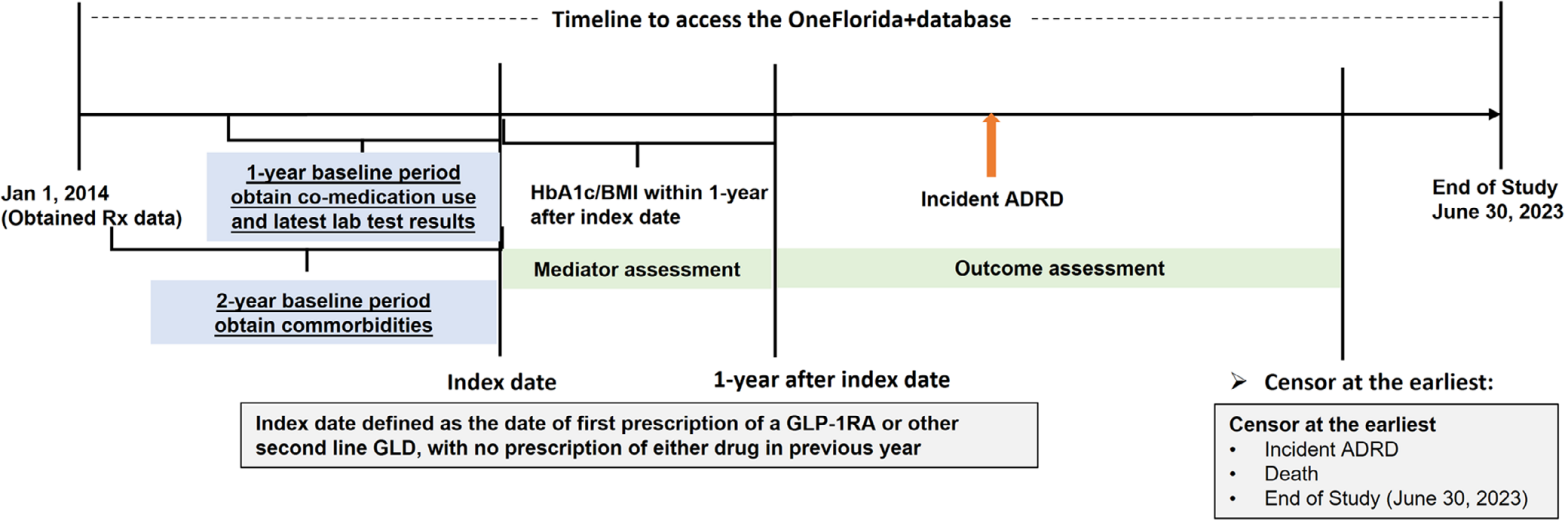
Overview of study design. Outcome assessment starts 1 year after the index date, and mediator information was obtained within 1 year after the index date to ensure that the mediator measurement preceded the outcome development.

We deployed an active comparator new user study design to mitigate the potential risk of confounding by indication and time‐related biases.^13^ The overview of the study design is shown in Figure 1. We utilized electronic health record data from the OneFlorida+ Clinical Research Consortium (from January 1, 2012, to June 30, 2023), a centralized repository containing patients’ demographics, diagnoses, medications, procedures, vital signs, and laboratory test results.^14^ This study was approved by the University of Florida Institutional Review Board (IRB202201196).

### 2.2 Study population

Eligible patients were those with T2D who initiated a GLP‐1RA or other second‐line glucose‐lowering drug (GLD) treatment (sulfonylurea, thiazolidinedione, dipeptidyl peptidase 4 inhibitor [DPP4i], α‐glucosidase inhibitor, or meglitinide) from January 2014 to June 2023. The cohort entry (index date) was the date of the first prescription for any of these medications, with no previous prescription of any drug in the preceding year. Patients were required to have at least 1‐year of follow‐up.

Exclusion criteria were: (1) age < 50 years; (2) prior diagnosis of ADRD; (3) use of United States Food and Drug Administration (FDA)‐approved anti‐dementia medications (such as donepezil, memantine, rivastigmine, galantamine, and aducanumab); (4) prior diagnosis of gestational diabetes, type 1 diabetes, or end‐stage renal disease/dialysis; (5) initiating both GLP‐1RA and other GLDs on the index date; (6) prior use of GLP‐1RA and other GLDs in previous year; (7) no physician visit in 2 years before index date; (8) no physician visit during follow‐up. The International Classification of Diseases (ICD) diagnosis codes are detailed in Table S1.

### 2.3 Treatment comparisons

We compared new users of GLP‐1RA with new users of other second‐line GLDs (Table S2). Medication use for GLP‐1RAs and other GLDs was measured based on provider prescription. GLP‐1RA use was defined by at least one prescription for exenatide, albiglutide, dulaglutide, liraglutide, lixisenatide, semaglutide, or tirzepatide. The comparator group included users of other second‐line GLDs, such as DPP4i, sulfonylureas, thiazolidinediones, α‐glucosidase inhibitors, and meglitinides. Insulin was excluded from the comparison due to its association with a longer duration of diabetes (a serious condition)^15^ and its significantly increased risk of all‐cause dementia.^16^, ^17^ Similarly, sodium‐glucose co‐transporter‐2 inhibitors (SGLT2is) were excluded from the comparator group because their known effects on both weight reduction and neuroprotection could confound our ability to isolate the potential mechanisms of GLP‐1RAs in reducing ADRD risk.^18^

### 2.4 Outcome definition and study follow‐up

The primary outcome was ADRD, which was identified using the 27 Chronic Conditions Warehouse (CCW) chronic condition algorithms.^19^ The diagnosis codes used for ADRD are detailed in Table S1. Patients were followed from the index date until the occurrence of ADRD, death, or the end of the study period (June 30, 2023), whichever occurred first (Figure 1). The outcome assessment started 1 year after the index date.

**RESEARCH IN CONTEXT**

1. **Systematic review**: A literature search was conducted using PubMed and previous studies indicated that glucagon‐like peptide‐1 receptor agonists (GLP‐1RAs) were associated with reductions in glycated hemoglobin (HbA1c) and body mass index (BMI), both risk factors for Alzheimer's disease and related dementias (ADRD). However, no studies have examined whether HbA1c and BMI reductions mediate the association between GLP‐1RAs and ADRD risk.
2. **Interpretation**: While our study observed significant reductions in HbA1c levels and BMI, as well as a lower ADRD risk associated with GLP‐1RA users compared to other glucose‐lowering drugs (GLDs) users, HbA1c and BMI reductions had minimal mediation effects on the association between GLP‐1RAs and ADRD risk.
3. **Future directions**: These results underscore the need for further investigation into the underlying mechanisms driving the protective effects of GLP‐1RAs against ADRD.

### 2.5 Mediator measure

As shown in Figure 1, HbA1c levels and BMI were continuously monitored up to 1 year after the index date to ensure that the mediator measurement preceded the outcome development. For mediation analysis, the HbA1c and BMI reduction, defined as the difference between baseline value and median value over this period, was calculated and used as mediators.

### 2.6 Statistical analysis

Patient characteristics were summarized using frequencies and percentages for categorical variables and means with standard deviations for continuous variables. Standardized mean differences (SMD) were used to assess the balance of baseline covariates between groups, with SMD < 0.1 indicating negligible differences.^20^

**FIGURE 2:**
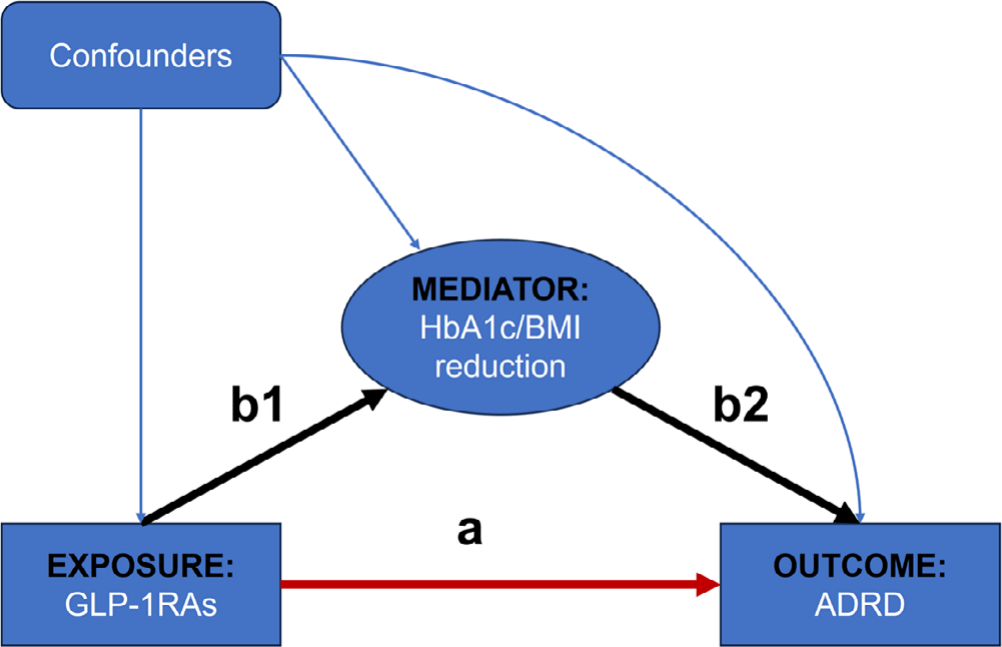
The directed acyclic graph of causal mediation analysis. The total effect of GLP‐1RAs on ADRD risk was decomposed into two distinct components: (1) nature direct effect (pathway a); and (2) natural indirect effect (pathway b1‐b2). The natural indirect effect refers to the effect of GLP‐1RAs on ADRD risk that is mediated by HbA1c or BMI reduction. The natural direct effect is the residual or direct effect of GLP‐1RAs on ADRD risk that is not mediated by HbA1c or BMI reduction. ADRD, Alzheimer's disease and related dementias; GLP‐1RAs, glucagon‐like peptide‐1 receptor agonists.

We estimated the adjusted mean difference (MD) with 95% confidence interval (CI) in HbA1c and BMI reduction between GLP‐1RAs and other GLDs using the multivariate generalized linear model. To estimate the extent to which the effect of GLP‐1RAs versus other GLDs on risk of ADRD, mediated by HbA1c or BMI reduction, we conducted causal mediation analysis under a counterfactual framework which relies on key assumptions, including no unmeasured confounding of the exposure‐outcome, mediator‐outcome, and exposure‐mediator relationships, as well as no exposure‐induced mediator‐outcome confounding.^21^ Compared to traditional mediation methods, causal mediation analysis offers advantages such as the ability to account for exposure‐mediator interactions, leading to more precise estimates of the natural direct effect, natural indirect effect, and total effect.^21^ The total effect of GLP‐1RAs on risk ADRD was decomposed into the natural direct effect and natural indirect effect^22^, ^23^ (Figure 2). The natural direct effect represents the effect of GLP‐1RAs on ADRD risk that is independent of HbA1c or BMI change, while the natural indirect effect represents the effect of GLP‐1RAs on ADRD risk that is mediated by changes in HbA1c or BMI. To estimate the direct and indirect effects of GLP‐1RAs on risk of ADRD, we fitted two models: (1) a multivariate Cox proportional hazards model was used to build the outcome model by regressing ADRD risk (outcome) against GLP1‐1RA (exposure), HbA1c or BMI reduction (mediator), and all baseline covariates; (2) a multivariate linear regression model was used to build the mediator model by regressing HbA1c or BMI reduction (mediator) against GLP‐1RA (exposure), and all baseline covariates.^24^ The regression parameters from the two models were used to calculate the adjusted hazard ratios (HRs) with 95% CIs for natural direct effect, natural indirect effect, and total effect, following the mathematical expression developed by Valeri and VanderWeele.^22^ We also computed the mediation effect measured by the percentage mediated, representing the percentage of the total effect that is mediated by the mediator. The percentage mediated was estimated using the formula: (Natural direct effect × (Natural indirect effect ‐1))/(Total effect ‐1).^25^ The baseline covariates included demographics (e.g., age, sex, and race/ethnicity), comorbidities (e.g., diabetic complications and hypertension), co‐medications (e.g., antihypertensives), baseline HbA1c levels, and baseline BMI, as listed in Table S3. Multiple imputation by chained equations including all baseline covariates was applied to address missing values of HbA1c (∼40%) and BMI(∼18%).^26^

### 2.7 Sensitivity analyses

Although our model accounted for potential confounders, unmeasured confounding may still influence the results. To address this, we calculated the E‐value, which quantifies the minimum strength of association an unmeasured confounder must have with both the exposure and the outcome to fully account for the observed association.^27^ A higher E‐value indicates stronger robustness of the findings, suggesting that the observed associations are less likely to be explained away by unmeasured confounding. Additionally, we conducted several sensitivity analyses to further evaluate the robustness of our findings. First, we used a complete case analysis including patients with HbA1c/BMI values at baseline and during the 1‐year follow‐up. Second, we used HbA1c and BMI reduction defined as the difference between baseline and mean values of HbA1c and BMI during the 1‐year follow‐up. Third, given that the risk of ADRD increases significantly with age, we conducted a separate mediation analysis among the older population (aged ≥ 65 years). Fourth, due to the complex and often non‐linear relationship between obesity and risk of ADRD, we performed a mediation analysis in patients with obesity. Fifth, we conducted an analysis that excluded patients with MCI at baseline, allowing us to examine the mediating pathways from normal cognitive function to ADRD. Sixth, we conducted a sensitivity analysis limited to patients receiving GLD monotherapy. Finally, we performed a traditional mediation analysis that did not account for exposure‐mediator interaction. All mediation analyses were undertaken using the CAUSALMED procedure in SAS v9.4 (SAS, Cary, NC, USA).

## 3 RESULTS

### 3.1 Study cohort

**FIGURE 3:**
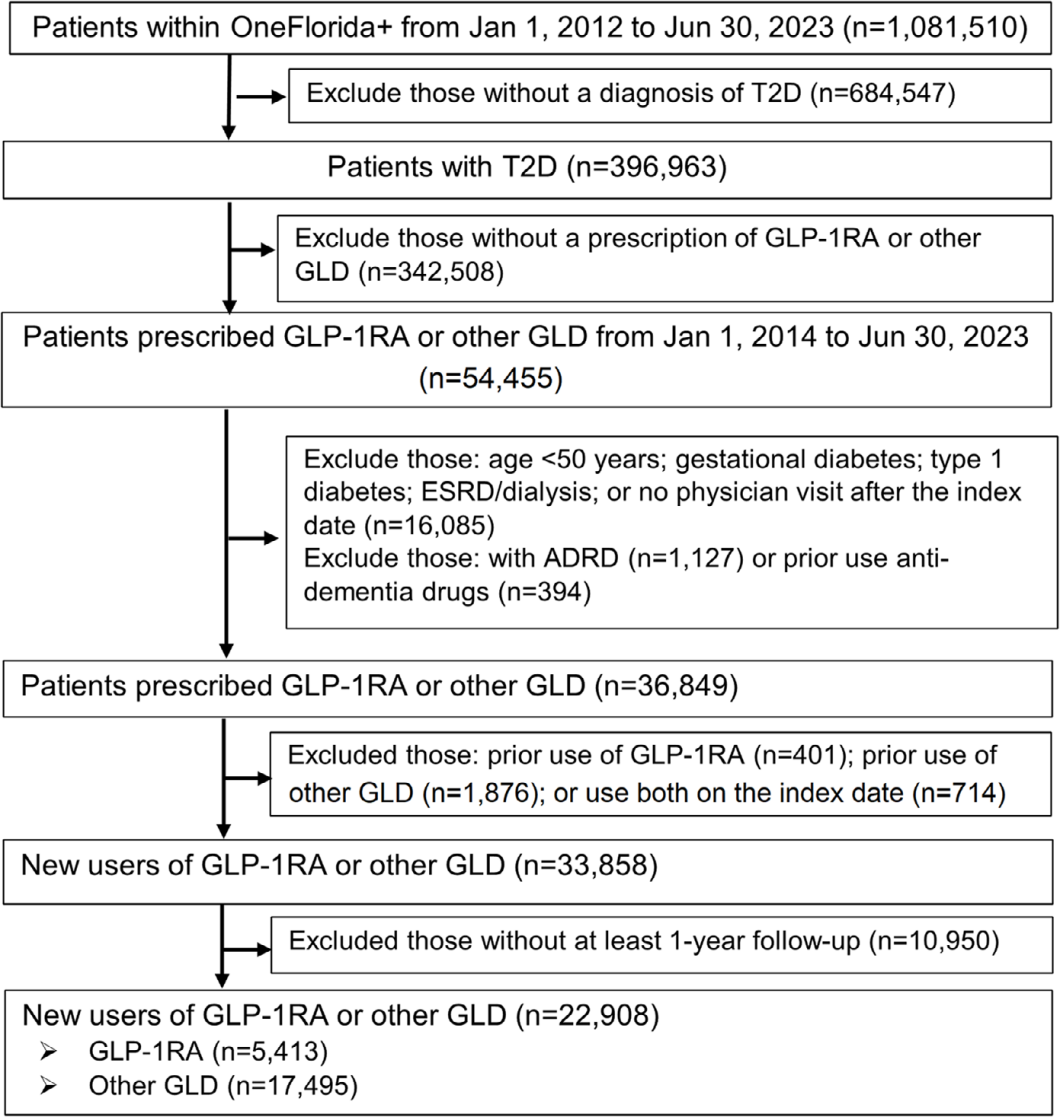
Flowchart of patient selection. ADRD, Alzheimer's Disease and Related Dementias; ESRD, end‐stage renal disease; GLP‐1RA, glucagon‐like peptide‐1 receptor agonists; GLD, glucose‐lowering drug; SGLT2i, sodium‐glucose cotransporter 2 inhibitors; T2D, type 2 diabetes.

Figure 3 shows the patient selection and process according to inclusion and exclusion. A total of 22,908 patients were included in the GLP‐1RA versus other GLD cohort. Among those, 5413 patients initiated GLP‐1RA, and 17,495 initiated other GLDs. The baseline characteristics of patients are presented in Table 1. Patients initiating GLP‐1RA therapy were younger (61.8 versus 65.5 years) and had higher BMI (35.4 versus 31.9 kg/m^2^) than other GLD initiators. Also, they had a higher proportion of diabetic retinopathy, diabetic neuropathy, hyperlipidemia, anxiety, sleep disorders, and obesity, but a lower prevalence of smoking and cardiovascular disease. Regarding medication use, GLP‐1RA initiators showed higher SGLT2i utilization but lower use of metformin, antihypertensives (e.g., angiotensin‐converting enzyme inhibitors, beta‐blockers, and calcium channel blockers), proton pump inhibitors, antipsychotics, benzodiazepines, opioid, and antiplatelet agents. During the follow‐up, 55 patients among 5,413 GLP‐1RA users developed ADRD (mean follow‐up: 3.69 years), and 458 among 17,495 other GLD users developed ADRD (mean follow‐up: 4.86 years).

**TABLE 1 alz70161-tbl-0001:** Baseline characteristics of patients included in GLP‐1RA versus other GLD cohort.

	GLP‐1RA versus other GLDs		
Characteristics	GLP‐1RA (*n* = 5,413)	Other GLDs (*n* = 17,495)	SMD
Age, years, mean(sd)	61.8 (7.9)	65.5 (9.4)	−0.426
Race/ethnicity			
Non‐Hispanic White	2451 (45.3%)	7988 (45.7%)	0.082
Non‐Hispanic Black	1422 (26.3%)	4806 (27.5%)	
Hispanics	1009 (18.6%)	2842 (16.2%)	
Other	531 (9.8%)	1859 (10.6%)	
Female	3131 (57.8%)	8854 (50.6%)	0.146
**Diabetes complications**			
Diabetic retinopathy	385 (7.1%)	698 (4.0%)	0.137
Diabetic neuropathy	810 (15.0%)	1893 (10.8%)	0.124
Peripheral vascular disease	410 (7.6%)	1454 (8.3%)	−0.027
Hypoglycemia	73 (1.3%)	102 (0.6%)	0.078
Hyperglycemic emergency	127 (2.3%)	380 (2.2%)	0.012
**Comorbidities**			
Ever smoking	50 (0.9%)	480 (2.7%)	−0.136
Mild cognitive impairment	22 (0.4%)	95 (0.5%)	−0.020
Parkinson's disease	22 (0.4%)	93 (0.5%)	−0.018
Cardiovascular disease	1138 (21.0%)	4617 (26.4%)	−0.126
Atrial fibrillation	315 (5.8%)	1513 (8.6%)	−0.109
Heart failure	407 (7.5%)	1515 (8.7%)	−0.042
Cerebrovascular disease	298 (5.5%)	1383 (7.9%)	−0.096
Hyperlipidemia	3258 (60.2%)	9643 (55.1%)	0.103
Traumatic brain injury	28 (0.5%)	123 (0.7%)	−0.024
Epilepsy/seizures	58 (1.1%)	206 (1.2%)	−0.010
Post‐traumatic stress disorder	35 (0.6%)	114 (0.7%)	−0.001
Bipolar disorder	71 (1.3%)	244 (1.4%)	−0.007
Schizophrenia	35 (0.6%)	132 (0.8%)	−0.013
Depression	792 (14.6%)	1911 (10.9%)	0.111
Anxiety	792 (14.6%)	1818 (10.4%)	0.128
Obsessive‐compulsive disorder	7 (0.1%)	23 (0.1%)	−0.001
Hypertension	3779 (69.8%)	12305 (70.3%)	−0.011
Chronic obstructive pulmonary disease	432 (8.0%)	1528 (8.7%)	−0.027
Chronic kidney disease	578 (10.7%)	2339 (13.4%)	−0.083
Periodontitis	30 (0.6%)	86 (0.5%)	0.009
Vitamin B12 deficiency	23 (0.4%)	52 (0.3%)	0.021
Asthma	502 (9.3%)	1260 (7.2%)	0.075
Inflammatory bowel disease	32 (0.6%)	95 (0.5%)	0.006
Anemia	892 (16.5%)	3414 (19.5%)	−0.079
Osteoporosis	177 (3.3%)	528 (3.0%)	0.014
Rheumatoid arthritis	1230 (22.7%)	3234 (18.5%)	0.105
Benign prostatic hyperplasia	264 (4.9%)	1025 (5.9%)	−0.044
Sleep disorder	1459 (27.0%)	3258 (18.6%)	0.200
Hearing impairment	226 (4.2%)	619 (3.5%)	0.033
Vision impairment	34 (0.6%)	104 (0.6%)	0.004
Alcohol use disorder	88 (1.6%)	417 (2.4%)	−0.054
Obesity	3879 (71.7%)	9062 (51.8%)	0.418
HIV/AIDS	54 (1.0%)	169 (1.0%)	0.003
Cataracts	595 (11.0%)	1465 (8.4%)	0.089
Glaucoma	269 (5.0%)	814 (4.7%)	0.015
Pancreatitis	39 (0.7%)	141 (0.8%)	−0.010
NAFLD	362 (6.7%)	757 (4.3%)	0.104
Thyroid disease	1014 (18.7%)	2585 (14.8%)	0.106
Hip/Pelvic fracture	10 (0.2%)	68 (0.4%)	−0.038
Cancer	530 (9.8%)	2112 (12.1%)	−0.073
**Co‐medications**			
ACEIs	1342 (24.8%)	5443 (31.1%)	−0.141
Beta‐blockers	1154 (21.3%)	5028 (28.7%)	−0.172
CCBs	1126 (20.8%)	4621 (26.4%)	−0.132
Diuretics	1477 (27.3%)	5181 (29.6%)	−0.052
Angiotensin receptor blockers	1152 (21.3%)	3561 (20.4%)	0.023
Statins	2579 (47.6%)	8902 (50.9%)	−0.065
Non‐statins for lowering lipid	417 (7.7%)	1351 (7.7%)	−0.001
NSAIDS	1120 (20.7%)	3537 (20.2%)	0.012
Proton pump inhibitors	1032 (19.1%)	4533 (25.9%)	−0.165
Antidepressant	570 (10.5%)	1857 (10.6%)	−0.003
Antipsychotics	185 (3.4%)	1017 (5.8%)	−0.114
Anti‐Parkinson agents	338 (6.2%)	1515 (8.7%)	−0.092
Benzodiazepines	787 (14.5%)	3508 (20.1%)	−0.146
Hormone replacement therapy	91 (1.7%)	218 (1.2%)	0.036
Oral steroids	1493 (27.6%)	5078 (29.0%)	−0.032
Opioid	1243 (23.0%)	6083 (34.8%)	−0.263
TNF inhibitors	21 (0.4%)	38 (0.2%)	0.031
Immunosuppressants	60 (1.1%)	250 (1.4%)	−0.029
Warfarin	84 (1.6%)	478 (2.7%)	−0.082
Direct oral anticoagulants	208 (3.8%)	908 (5.2%)	−0.065
Aspirin	702 (13.0%)	4025 (23.0%)	−0.264
Non‐aspirin antiplatelet agents	278 (5.1%)	1559 (8.9%)	−0.148
**Other GLDs**			
Insulin	2153 (39.8%)	6270 (35.8%)	0.081
Metformin	2267 (41.9%)	8113 (46.4%)	−0.091
SGLT2is	202 (3.7%)	200 (1.1%)	0.169
**Other**			
Baseline HbA1c, %	8.3 (2.1)	8.0 (2.2)	0.131
Baseline BMI, kg/m^2^	35.2 (7.1)	31.7 (6.7)	0.497
Baseline systolic blood pressure, mmHg	133.2 (16.6)	133.1 (16.9)	0.010
Baseline diastolic blood pressure, mmHg	75.8 (9.7)	74.5 (10.2)	0.131

Abbreviations: ACEIs, angiotensin‐converting‐enzyme inhibitors; BMI, body mass index; CCB, calcium channel blocker; GLD, glucose‐lowering drug; GLP‐1RA, glucagon‐like peptide‐1 receptor agonists; HbA1c, hemoglobin A1C; HIV/AIDS, human immunodeficiency virus/acquired immunodeficiency syndrome; NAFLD, nonalcoholic fatty liver disease; NSAIDS, nonsteroidal anti‐inflammatory drugs; SGLT2i, sodium‐glucose cotransporter 2 inhibitors; SMD, Standardized mean differences; TNF inhibitors, tumor necrosis factor inhibitors.

### 3.2 GLP‐1RAs, HbA1c/BMI reduction, and ADRD risk

After adjusting[Table alz70161-tbl-0001], [Table alz70161-tbl-0002] for all baseline covariates, we observed significant reductions in both HbA1c levels (adjusted MD, −0.16%; 95% CI, −0.20 to −0.12) and BMI (adjusted MD, −0.23 kg/m^2^; 95% CI, −0.31 to −0.15) among GLP‐1RA users compared to other GLD users. The results of causal mediation analysis are presented in Table 2. The adjusted HR for the total effect of GLP‐1RAs versus other GLDs on ADRD risk was 0.74 (95% CI, 0.52 to 0.96), suggesting a lower risk of ADRD associated with GLP‐1RAs compared to other GLDs. When decomposing the total effect into natural direct effect and natural indirect effect using HbA1c reduction as the mediator, the adjusted HRs were 0.73 (95% CI, 0.51 to 0.95) for natural direct effect and 1.02 (95% CI, 0.99 to 1.04) for natural indirect effect. Similarly, when BMI reduction was used as the mediator, the adjusted HRs were 0.75 (95% CI, 0.53 to 0.97) for natural direct effect and 1.02 (95% CI, 0.99 to 1.04) for natural indirect effect. Both HbA1c and BMI reductions had minimal mediation effects on the association between GLP‐1RAs and decreased risk of ADRD, with proportion mediated value of −5.21% (−13.56 to 3.13) and −5.03% (−14.09 to 4.02), respectively.

**TABLE 2 alz70161-tbl-0002:** The total effect, natural direct effect, natural indirect effect, and “proportion mediated” estimates of GLP‐1RAs on risk of Alzheimer's disease and related dementias, mediated by HbA1c or BMI reduction, after adjusting for baseline covariates.

Parameter	Totel effect, aHR (95% CI)^a^	Natural direct effect, aHR (95% CI)^a^	Natural indirect effect, aHR (95% CI)^a^	Proportion mediated, % (95% CI)^a^
HbA1c reduction	0.74 (0.52,0.96)	0.73 (0.51,0.95)	1.02 (0.99,1.04)	−5.21 (−13.56,3.13)
BMI reduction	0.76 (0.54, 0.99)	0.75 (0.53,0.97)	1.02 (0.99,1.04)	−5.03 (−14.09,4.02)

Abbreviations: aHR, adjusted hazard ratio; BMI, body mass index; CI, confidence interval; GLP‐1RA, glucagon‐like peptide‐1 receptor agonists; HbA1c, glycated hemoglobin.

^a^ Adjusted for baseline covariates outlined in Table 1.

### 3.3 Sensitivity analyses

To evaluate the robustness of our model against potential unmeasured confounding, we calculate the E‐value. The E‐value was 1.60 for HbA1c reduction and 1.56 for BMI reduction, indicating the observed association could be explained away if an unmeasured confounder had an HR association greater than 1.60 and 1.56 with both exposure and outcome. Additionally, to further validate our primary findings, we conducted a series of sensitivity analyses, with detailed results presented in Table S4. The results from sensitivity analyses were consistent with the primary analysis, supporting the robustness of our primary findings.

## 4 DISCUSSION

Our study employed causal mediation analysis to investigate the roles of HbA1c and BMI reductions as potential mediators in the association of GLP‐1RAs with risk of ADRD among adults with T2D. We found that GLP‐1RAs were significantly associated with a decreased risk of ADRD compared to other GLDs. However, the causal mediation analysis found that the protective effect of GLP‐1RAs on ADRD risk was primarily direct, with HbA1c and BMI reductions playing minimal roles in the mediation of the association. These findings, which remained consistent across sensitivity analyses, suggest that GLP‐1RAs may protect against ADRD through mechanisms largely independent of their effects on HbA1c and BMI reductions.

This study found a lower risk of ADRD associated with GLP‐1RAs than other GLDs. Specifically, GLP‐1RAs were associated with a 26% reduction in ADRD risk. These findings align with and extend previous research suggesting neuroprotective effects of GLP‐1RAs.^3^, ^28^ Our study adds to this body of evidence by employing a more rigorous causal mediation analysis approach to explore potentially underlying mechanistic pathways. We found that the protective effects of GLP‐1RAs on ADRD risk were minimally mediated through their effects on HbA1c or BMI reduction. These findings suggest that GLP‐1RAs may protect against ADRD through mechanisms beyond their known effects on glycemic control and body weight. The minimal mediation through HbA1c and BMI despite the improvement in glycemic control and weight loss suggests that the relationship between GLP‐1RAs, glucose regulation, weight loss, and cognitive outcomes may be more complex than previously thought.

The lack of substantial mediation through HbA1c and BMI reduction suggests that GLP‐1RAs may exert their neuroprotective effects through alternative pathways. Several potential mechanisms warrant further investigation: (a) direct effects on brain insulin signaling: GLP‐1RAs have been shown to cross the blood‐brain barrier^29^ and may directly modulate insulin signaling in the brain,^18^, ^30^, ^31^ which is increasingly recognized as important in AD pathogenesis.^32^ (b) reduction of neuroinflammation: GLP‐1RAs have anti‐inflammatory properties that could protect against neurodegenerative processes.^18^, ^33^, ^34^ (c) modulation of amyloid metabolism: Some studies have suggested that GLP‐1RAs may influence amyloid processing and clearance in the brain.^35^, ^36^ (d) effects on mitochondrial function and oxidative stress: GLP‐1RAs have been associated with improvements in mitochondrial function and oxidative stress,^18^, ^37^ which could protect against neuronal damage. (e) improve vascular outcomes: GLP‐1RAs have been associated with improved cardiovascular outcomes,^38^, ^39^ which may contribute to better cognitive outcomes given the strong link between vascular health and cognitive function.^40^ Future mechanistic studies, including neuroimaging and biomarker analyses, will be crucial to elucidate these potential pathways.

Our results support the potential use of GLP‐1RAs as part of a strategy to reduce ADRD risk in patients with T2D. The benefits appear to extend beyond their effects on glycemic control and body weight, suggesting that both drugs may have value even in patients with well‐controlled diabetes. This could influence clinical decision‐making, particularly for patients at high risk of cognitive decline. However, it is important to note that our study does not provide definitive evidence for using GLP‐1RAs solely for cognitive protection. The decision to prescribe these drugs should still be based primarily on their approved indications for diabetes management and cardiovascular risk reduction. Given the limited mediation by HbA1c and BMI, future studies should explore other potential mediators of the protective effects of GLP‐1RAs, such as cardiovascular and renal outcomes, markers of inflammation (e.g., C‐reactive protein and interleukin‐6), and biomarkers of AD pathology (e.g., amyloid and tau in cerebrospinal fluid)

Several limitations should be considered when interpreting our findings, despite our use of causal mediation analysis provided a more nuanced understanding of the relationships between GLP‐1RAs, metabolic factors, and ADRD risk. First, a substantial proportion of missing data for HbA1c and BMI is the major limitation of this study. Specifically, approximately 40% of HbA1c values and 18% of BMI measurements were missing. While multiple imputation by chained equations was applied to address the missing values, this level of missing data introduces uncertainty into our analyses and potentially limits the robustness of our conclusions. However, our complete case analysis yielded similar results, supporting the robustness of our findings. Second, despite employing advanced statistical methods, the observational nature of our study design inherently limits causal inference. Residual confounding (e.g., severity of diabetes) remains a possibility despite our comprehensive adjustment for baseline covariates. This may challenge the key assumptions of causal mediation analysis regarding unmeasured confounding. However, the E‐value of 1.60 suggests moderate robustness, indicating that only a confounder with a sufficiently strong association with both the exposure and outcome could fully explain the observed association. Third, the relatively short follow‐up period may limit our ability to detect long‐term effects on ADRD risk. Given the chronic nature of ADRD development, future investigations would benefit from extended observation periods to better characterize these temporal relationships. Fourth, although we used CCW algorithms to identify ADRD cases, potential misclassification is possible, particularly for early or mild cases. Such misclassification could potentially bias our results. Additionally, MCI is often underdiagnosed in administrative data, which may limit the impact of the sensitivity analysis excluding patients with MCI. This underdiagnosis could lead to an overestimation of the effects of GLP‐1RAs on the progression from normal cognitive function to ADRD. Fifth, our study was conducted using data from OneFlorida+, which may not fully represent the broader U.S. population or populations in other countries. The generalizability of our findings to other demographic groups or healthcare settings should be considered with caution. Sixth, the use of GLP‐1RAs and other GLDs was determined based on provider prescriptions, which limited the causal inference, as it could not account for whether the medications were actually dispensed by the pharmacy or adhered to by the patients.

This study provides evidence for a protective effect of GLP‐1RAs against ADRD in adults with T2D, largely independent of their effects on HbA1c and BMI. These findings underscore the potential of GLP‐1RAs as part of a multifaceted approach to ADRD prevention in people with T2D and highlight the need for further research into their mechanisms of action and long‐term cognitive effects.

## CONFLICT OF INTEREST STATEMENT

All authors declare no conflict of interest. Author disclosures are available in the supporting information.

## CONSENT STATEMENT

This study was approved by the University of Florida Institutional Review Board (IRB202201196). This is a secondary analysis and obtaining informed consent for this study was not necessary.

## Supporting information



Supporting Information

Supporting Information

## References

[1] 2023 Alzheimer's disease facts and figures. Alzheimers Dement J Alzheimers Assoc. 2023;19:1598‐1695. 10.1002/alz.13016 36918389

[2] 2024 Alzheimer's disease facts and figures. Alzheimers Dement J Alzheimers Assoc. 2024;20:3708‐3821. 10.1002/alz.13809 PMC11095490 38689398

[3] Tang H , Shao H , Shaaban CE , et al. Newer glucose‐lowering drugs and risk of dementia: a systematic review and meta‐analysis of observational studies. J Am Geriatr Soc. 2023;71(7):2096‐2106. 36821780 10.1111/jgs.18306 PMC10363181

[4] He D , Aleksic S . Is it time to repurpose geroprotective diabetes medications for prevention of dementia? J Am Geriatr Soc. 2023;71(7):2041‐2045. 37227136 10.1111/jgs.18405 PMC10524156

[5] Yao H , Zhang A , Li D , et al. Comparative effectiveness of GLP‐1 receptor agonists on glycaemic control, body weight, and lipid profile for type 2 diabetes: systematic review and network meta‐analysis. BMJ. 2024;384:e076410. 38286487 10.1136/bmj-2023-076410 PMC10823535

[6] Teo YH , Teo YN , Syn NL , et al. Effects of sodium/glucose cotransporter 2 (SGLT2) inhibitors on cardiovascular and metabolic outcomes in patients without diabetes mellitus: a systematic review and meta‐analysis of randomized‐controlled trials. J Am Heart Assoc. 2021;10:e019463. 33625242 10.1161/JAHA.120.019463 PMC8174267

[7] Crane PK , Walker R , Hubbard RA , et al. Glucose levels and risk of dementia. N Engl J Med. 2013;369:540‐548. 23924004 10.1056/NEJMoa1215740 PMC3955123

[8] Ramirez A , Wolfsgruber S , Lange C , et al. Elevated HbA1c is associated with increased risk of incident dementia in primary care patients. J Alzheimers Dis JAD. 2015;44:1203‐1212. 25524954 10.3233/JAD-141521

[9] Yaffe K , Falvey C , Hamilton N , et al. Diabetes, glucose control, and 9‐year cognitive decline among older adults without dementia. Arch Neurol. 2012;69:1170‐1175. 22710333 10.1001/archneurol.2012.1117 PMC3752423

[10] Wang F , Luo J , Ding D , et al. Elevated fasting blood glucose level increases the risk of cognitive decline among older adults with diabetes mellitus: the shanghai aging study. J Alzheimers Dis JAD. 2019;67:1255‐1265. 30689569 10.3233/JAD-180662

[11] Emmerzaal TL , Kiliaan AJ , Gustafson DR . 2003‐2013: A decade of body mass index, Alzheimer's disease, and dementia. J Alzheimers Dis. 2015;43:739‐755. 25147111 10.3233/JAD-141086

[12] Imai K , Keele L , Tingley D . A general approach to causal mediation analysis. Psychol Methods. 2010;15:309‐334. 20954780 10.1037/a0020761

[13] Lund JL , Richardson DB , Stürmer T . The active comparator, new user study design in pharmacoepidemiology: Historical foundations and contemporary application. Curr Epidemiol Rep. 2015;2:221. 26954351 10.1007/s40471-015-0053-5 PMC4778958

[14] Hogan WR , Shenkman EA , Robinson T , et al. The OneFlorida Data Trust: a centralized, translational research data infrastructure of statewide scope. J Am Med Inform Assoc. 2022;29:686‐693. 34664656 10.1093/jamia/ocab221 PMC8922180

[15] Weiner JZ , Gopalan A , Mishra P , et al. Use and discontinuation of insulin treatment among adults aged 75 to 79 years with type 2 diabetes. JAMA Intern Med. 2019;179:1633‐1641. 31545376 10.1001/jamainternmed.2019.3759 PMC6763990

[16] Zhou J‐B , Tang X , Han M , Yang J , Simó R . Impact of antidiabetic agents on dementia risk: A Bayesian network meta‐analysis. Metabolism. 2020;109:154265. 32446679 10.1016/j.metabol.2020.154265

[17] Alkabbani W , Maxwell CJ , Marrie RA , Tyas SL , Lega IC , Gamble JM . Insulin use in type 2 diabetes and the risk of dementia: a comparative population‐based cohort study. Diabetes Care dc 2023;230222:1492‐1500. doi:10.2337/dc23-0222 37315211

[18] Pawlos A , Broncel M , Woźniak E , Gorzelak‐Pabiś P . Neuroprotective effect of SGLT2 inhibitors. Molecules. 2021;26:7213. 34885795 10.3390/molecules26237213 PMC8659196

[19] Chronic Conditions.Chronic Conditions Data Warehouse. https://www2.ccwdata.org/web/guest/condition-categories-chronic

[20] Austin PC . Balance diagnostics for comparing the distribution of baseline covariates between treatment groups in propensity‐score matched samples. Stat Med. 2009;28:3083‐3107. 19757444 10.1002/sim.3697 PMC3472075

[21] Li Y , Yoshida K , Kaufman JS , Mathur MB . A brief primer on conducting regression‐based causal mediation analysis. Psychol Trauma Theory Res Pract Policy. 2023;15:930‐938. 10.1037/tra0001421 PMC10368791 36701540

[22] Valeri L , VanderWeele TJ . Mediation analysis allowing for exposure‐mediator interactions and causal interpretation: Theoretical assumptions and implementation with SAS and SPSS macros. Psychol Methods. 2013;18:137. 23379553 10.1037/a0031034 PMC3659198

[23] VanderWeele TJ , Vansteelandt S . Mediation analysis with multiple mediators. Epidemiol Methods. 2014;2:95. 25580377 10.1515/em-2012-0010 PMC4287269

[24] VanderWeele TJ . A unification of mediation and interaction: a 4‐way decomposition. Epidemiol Camb Mass. 2014;25:749‐761. 10.1097/EDE.0000000000000121 PMC4220271 25000145

[25] VanderWeele TJ , Vansteelandt S . Odds ratios for mediation analysis for a dichotomous outcome. Am J Epidemiol. 2010;172:1339‐1348. 21036955 10.1093/aje/kwq332 PMC2998205

[26] Jakobsen JC , Gluud C , Wetterslev J , Winkel P . When and how should multiple imputation be used for handling missing data in randomised clinical trials – a practical guide with flowcharts. BMC Med Res Methodol. 2017;17:162. 29207961 10.1186/s12874-017-0442-1 PMC5717805

[27] VanderWeele TJ , Ding P . Sensitivity analysis in observational research: introducing the E‐Value. Ann Intern Med. 2017;167:268‐274. 28693043 10.7326/M16-2607

[28] Tang B , Sjölander A , Wastesson JW , et al. Comparative effectiveness of glucagon‐like peptide‐1 agonists, dipeptidyl peptidase‐4 inhibitors, and sulfonylureas on the risk of dementia in older individuals with type 2 diabetes in Sweden: an emulated trial study. eClinicalMedicine. 2024;73:102689. 10.1016/j.eclinm.2024.102689 PMC11490655 39429814

[29] Dong M , Wen S , Zhou L . The Relationship between the blood‐brain‐barrier and the central effects of glucagon‐like peptide‐1 receptor agonists and sodium‐glucose cotransporter‐2 inhibitors. Diabetes Metab Syndr Obes Targets Ther. 2022;15:2583‐2597. 10.2147/DMSO.S375559 PMC9417299 36035518

[30] Nowell J , Blunt E , Edison P . Incretin and insulin signaling as novel therapeutic targets for Alzheimer's and Parkinson's disease. Mol Psychiatry. 2023;28:217‐229. 36258018 10.1038/s41380-022-01792-4 PMC9812772

[31] Rizzo MR , Di Meo I , Polito R , et al. Cognitive impairment and type 2 diabetes mellitus: focus of SGLT2 inhibitors treatment. Pharmacol Res. 2022;176:106062. 35017046 10.1016/j.phrs.2022.106062

[32] Akhtar A , Sah SP . Insulin signaling pathway and related molecules: role in neurodegeneration and Alzheimer's disease. Neurochem Int. 2020;135:104707. 32092326 10.1016/j.neuint.2020.104707

[33] Diz‐Chaves Y , Mastoor Z , Spuch C , González‐Matías LC , Mallo F . Anti‐Inflammatory effects of GLP‐1 receptor activation in the brain in neurodegenerative diseases. Int J Mol Sci. 2022;23:9583. 36076972 10.3390/ijms23179583 PMC9455625

[34] Yoon G , Kim Y‐K , Song J . Glucagon‐like peptide‐1 suppresses neuroinflammation and improves neural structure. Pharmacol Res. 2020;152:104615. 31881271 10.1016/j.phrs.2019.104615

[35] Li Y , Duffy KB , Ottinger MA , et al. GLP‐1 receptor stimulation reduces amyloid‐β peptide accumulation and cytotoxicity in cellular and animal models of Alzheimer's disease. J Alzheimers Dis JAD. 2010;19:1205‐1219. 20308787 10.3233/JAD-2010-1314 PMC2948479

[36] McClean PL , Gault VA , Harriott P , Hölscher C . Glucagon‐like peptide‐1 analogues enhance synaptic plasticity in the brain: a link between diabetes and Alzheimer's disease. Eur J Pharmacol. 2010;630:158‐162. 20035739 10.1016/j.ejphar.2009.12.023

[37] Nuamnaichati N , Mangmool S , Chattipakorn N , Parichatikanond W . Stimulation of GLP‐1 receptor inhibits Methylglyoxal‐induced mitochondrial dysfunctions in H9c2 cardiomyoblasts: potential role of Epac/PI3K/Akt pathway. Front Pharmacol. 2020;11:805. 32547400 10.3389/fphar.2020.00805 PMC7274035

[38] Nelson AJ , Pagidipati NJ , Aroda VR , et al. Incorporating SGLT2i and GLP‐1RA for cardiovascular and kidney disease risk reduction: call for action to the cardiology community. Circulation. 2021;144:74‐84. 34228476 10.1161/CIRCULATIONAHA.121.053766

[39] Kristensen SL , Rørth R , Jhund PS , et al. Cardiovascular, mortality, and kidney outcomes with GLP‐1 receptor agonists in patients with type 2 diabetes: a systematic review and meta‐analysis of cardiovascular outcome trials. Lancet Diabetes Endocrinol. 2019;7:776‐785. 31422062 10.1016/S2213-8587(19)30249-9

[40] Samieri C , Perier M‐C , Gaye B , et al. Association of cardiovascular health level in older age with cognitive decline and incident dementia. JAMA. 2018;320:657‐664. 30140876 10.1001/jama.2018.11499 PMC6142948

