# Supplementary material for: Glycated hemoglobin and body mass index as mediators of GLP‐1RAs and Alzheimer's disease and related dementias in patients with type 2 diabetes
Source: Alzheimers Dement. 2025 Apr 10;21(4):e70161. doi: 10.1002/alz.70161 (PMC11982930; doi:10.1002/alz.70161)
Supplement: Supplementary file 1 — Supporting Information [file ALZ-21-e70161-s001.docx]

**Supplemental materials**

**Table S1.** Codes used for outcome definition and key variables.

| Outcome/key variables | ICD-9 | ICD-10 |
| --- | --- | --- |
| Alzheimer's disease and related dementias | 331.0, 331.11, 331.19, 331.2, 331.7, 290.0, 290.10, 290.11, 290.12, 290.13, 290.20, 290.21, 290.3, 290.40, 290.41, 290.42, 290.43, 294.0, 294.10, 294.11, 294.20, 294.21, 294.8, 797 | F01.50, F01.51, F02.80, F02.81, F03.90, F03.91, F04, F05, F06.1, F06.8, G13.8, G30.0, G30.1, G30.8, G30.9, G31.01, G31.09, G31.1, G31.2, G94, R41.81, R54 |
| Type 2 diabetes | 250.x0; 250.x2 | E11 |
| Type 1 diabetes | 250.x1; 250.x3 | E10 |
| Gestational diabetes | 648.0x | O24 |
| End-Stage Renal Disease (ESRD) | 346 | G43 |
| Dialysis | 38.95, 39.27, 39.42, 39.95, 54.98, V45.1, V45.11, V45.12, V56.0, V56.1, V56.2, V56.31, V56.32,  V56.8 | T81.502, T81.502A, T81.502D, T81.502S, T82.4, T82.41, T82.41XA, T82.41XD, T82.41XS, T82.42, T82.42XA, T82.42XD, T82.42XS, T82.43, T82.43XA, T82.43XD, T82.43XS, T82.49, T82.49XA, T82.49XD, T82.49XS, T85.61, T85.611, T85.611A, T85.611D, T85.611S, T85.621, T85.621A, T85.621D, T85.621S, T85.622, T85.622A, T85.622D, T85.622S, T85.631, T85.631A, T85.631D, T85.631S, T85.691, T85.691A, T85.691D, T85.691S, Z49.0, Z49.01, Z49.02, Z49.3, Z49.31, Z49.32, Z91.15, Z99.2 |
| Antidementia drugs | donepezil, memantine, rivastigmine, galantamine, and aducanumab | |

**Table S2.** Drugs of interest.

| GLP-1RAs | GLP-1RAs | Exenatide; Albiglutide; Dulaglutide; Liraglutide; Lixisenatide; Semaglutide; Tirzepatide |
| --- | --- | --- |
| Other second-line GLDs | DPP4is | Sitagliptin; Saxagliptin; Alogliptin; Linagliptin |
|  | Biguanides | Metformin |
|  | Sulfonylureas | Glimepiride; Glyburide; Chlorpropamide; Glipizide; Tolbutamide; Tolazamide |
|  | Thiazolidinediones | Pioglitazone; Rosiglitazone |
|  | Meglitinides | Repaglinide; Nateglinide |
|  | α-glucosidase inhibitors | Miglitol; Acarbose |
| DPP4is, dipeptidyl peptidase 4 inhibitors; GLP-1RAs, glucagon-like peptide-1 receptor agonists; GLDs, glucose-lowering drugs. | | |

**Table S3.** Baseline covariates

| Demographic characteristics (index date) | Age, sex, race/ethnicity |
| --- | --- |
| Diabetes complications(2 years before or on the index date) | diabetes retinopathy, diabetic neuropathy, peripheral vascular disease, hypoglycemia, or hyperglycemic emergency |
| Comorbidities (2 years before or on the index date) | Ever smoking,  Mild cognitive impairment,  Parkinson’s disease,  Cardiovascular disease,  Atrial fibrillation,  Heart failure,  Cerebrovascular disease,  Hyperlipidemia,  Traumatic brain injury,  Epilepsy/Seizures,  Post-traumatic stress disorder,  Bipolar,  Schizophrenia,  Depression,  Anxiety,  Obsessive-compulsive disorder,  Hypertension,  Chronic obstructive pulmonary disease,  Chronic kidney disease,  Periodontitis,  Vitamin B12 deficiency,  Asthma,  Inflammatory bowel disease,  Anemia,  Osteoporosis,  Rheumatoid arthritis,  Benign prostatic hyperplasia,  Sleep disorder,  Hearing impairment,  Vision impairment,  Alcohol use disorder,  Obesity,  Human immunodeficiency virus/acquired immunodeficiency syndrome,  Cataracts,  Glaucoma,  Pancreatitis,  Nonalcoholic fatty liver disease,  Thyroid disease,  Hip/Pelvic fracture,  Cancer |
| Medications (1 year before or on the index date) | Angiotensin-converting-enzyme inhibitors,  Beta-blockers,  Calcium channel blockers,  Diuretics,  Angiotensin receptor blockers,  Statins,  Non-statins for lowering lipid,  nonsteroidal anti-inflammatory drugs,  Proton pump inhibitors,  Antidepressant,  Antipsychotics,  Anti-Parkinson agents,  Benzodiazepines,  Hormone replacement therapy,  Oral steroids,  Opioid,  Tumor Necrosis Factor inhibitors,  Immunosuppressants,  Warfarin,  Direct oral anticoagulants,  Aspirin,  Non-aspirin antiplatelet agents,  Insulin,  Metformin, |
| Others (most recent value in the previous year) | HbA1c, body mass index |

**Table S4.** Sensitivity analyses of total effect (TE), natural direct effect (NDE), natural indirect effect (NIE), and “proportion mediated” (PM) estimates of GLP-1RAs on Alzheimer's disease and related dementias (ADRD) risk, mediated by HbA1c or BMI reduction, after adjusting for baseline covariates.

|  | **Totel effect, aHR (95% CI) ^a^** | **Natural direct effect, aHR (95% CI)^a^** | **Natural indirect effect, aHR (95% CI)^a^** | **Proportion mediated, (95% CI), %^a^** |
| --- | --- | --- | --- | --- |
| **Complete case analysis** | | | | |
| HbA1c reduction | 0.75(0.40,1.11) | 0.76(0.40,1.13) | 0.99(0.94,1.04) | 3.37(-13.19,19.92) |
| BMI reduction | 0.58(0.34,0.81) | 0.57(0.34,0.81) | 1.01(0.96,1.06) | -1.23(-7.61, 5.15) |
| **Using mean HbA1c and BMI reduction** | | | | |
| HbA1c reduction | 0.74(0.52,0.96) | 0.72(0.50,0.94) | 1.02(1.00,1.05) | -5.78 (-14.69, 3.12) |
| BMI reduction | 0.76(0.53,0.98) | 0.75(0.52,0.97) | 1.02(1.00,1.04) | -5.94 (-715.93, 4.06) |
| **Among patients with obesity** | | | | |
| HbA1c reduction | 0.74(0.47,1.02) | 0.72(0.46,0.99) | 1.03(1.00,1.06) | -8.06 (-21.85,5.73) |
| BMI reduction | 0.79(0.51,1.07) | 0.77(0.50,1.05) | 1.02(0.99,1.05) | -8.17(-25.57,9.23) |
| **Among older patients** | | | | |
| HbA1c reduction | 0.72(0.44,1.00) | 0.71(0.43,0.98) | 1.01(0.99,1.04) | -3.62(-10.93, 3.69) |
| BMI reduction | 0.76(0.47,1.05) | 0.76(0.47,1.05) | 1.00(0.97,1.03) | -0.47(-11.07,10.14) |
| **Among patients without mild cognitive impairment** | | | | |
| HbA1c reduction | 0.73(0.51,0.96) | 0.72(0.50,0.94) | 1.02(0.99,1.04) | -4.23(-11.81,3.34) |
| BMI reduction | 0.75(0.53,0.98) | 0.74(0.52,0.97) | 1.01(0.99,1.04) | -4.18(-12.39,4.04) |
| **GLP-1RA monotherapy use** | | | | |
| HbA1c reduction | 0.41(0.16,0.66) | 0.41(0.16,0.66) | 1.00(0.95,1.06) | -0.09(-3.91,3.72) |
| BMI reduction | 0.42(0.16,0.67) | 0.41(0.15,0.66) | 1.02(0.96,1.09) | -1.55(-6.26,3.16) |
| **Without accounting for exposure-mediator interaction** | | | | |
| HbA1c reduction | 0.76(0.53,0.98) | 0.76(0.53,0.98) | 1.00(0.99,1.01) | -0.12(-3.72,3.47) |
| BMI reduction | 0.76(0.54,0.99) | 0.75(0.53,0.98) | 1.01(1.00,1.02) | -4.22(-10.27,1.83） |

^a^adjusted for baseline covariates outlined in Table 1.

HbA1c, glycated hemoglobin; BMI, body mass index; HR, hazard ratio; CI, confidence interval.
